# Melioidosis vaccines: recent advances and future directions

**DOI:** 10.3389/fimmu.2025.1582113

**Published:** 2025-06-24

**Authors:** Sineenart Sengyee, Sarah B. Weiby, Ivory T. Rok, Mary N. Burtnick, Paul J. Brett

**Affiliations:** ^1^ Department of Microbiology and Immunology, University of Nevada, Reno School of Medicine, Reno, NV, United States; ^2^ Department of Microbiology and Immunology, Faculty of Tropical Medicine, Mahidol University, Bangkok, Thailand

**Keywords:** *Burkholderia pseudomallei*, *Burkholderia mallei*, melioidosis, glanders, vaccine, review

## Abstract

Melioidosis, caused by the Gram-negative bacterium *Burkholderia pseudomallei*, is a severe infectious disease that is responsible for a significant amount of morbidity and mortality in endemic areas. While the majority of melioidosis cases occur in Southeast Asia, South Asia and Northern Australia, the disease is being increasingly recognized across tropical and subtropical regions worldwide. Due to diagnostic and treatment challenges as well as the potential misuse of *B. pseudomallei* as a biothreat agent, an effective vaccine is critically needed. Over the years, numerous different strategies have been explored to develop melioidosis vaccines. Based on the choice of protective antigens, many of the resulting candidates would also be predicted to provide some level of protection against *Burkholderia mallei*, the etiologic agent of glanders. In this review, we examine the different approaches that have recently been used to develop melioidosis vaccine candidates, highlighting both traditional and emerging vaccine platform technologies. Using these approaches, several promising melioidosis and glanders candidates have been identified with pre-clinical animal studies providing valuable insights into the immunogenic and protective capacities of these potential vaccines. Collectively, this review summarizes recent advancements in melioidosis vaccine research and highlights critical findings that will help guide a path toward the development of a safe, effective and affordable vaccine to combat disease caused by *B. pseudomallei*.

## 1 Introduction

*Burkholderia pseudomallei* is the causative agent of melioidosis, a severe infectious disease that is known to be endemic in 45 countries in Southeast Asia, South Asia, the Middle East, Africa, Central America, and South America (1, 2). Regions where the disease is being detected are increasing and models predict an additional 34 countries where it is likely present but yet to be reported (2). Notably, there have been recent reports of locally acquired cases of melioidosis in the U.S. making this disease more widespread than previously appreciated (3). In a study published in 2016, the estimated incidence of disease was ~165,000 cases per year worldwide with ~89,000 associated deaths (2). Melioidosis has a wide range of clinical manifestations that vary from chronic localized infections to acute pneumonias and fulminant sepsis. As a result of poor diagnostics, a lack of clinical and laboratory expertise in endemic regions, and misdiagnosis due to diverse clinical presentations, the disease is severely underreported (4–10).

*B. pseudomallei* is a motile, facultative-intracellular, Gram-negative pathogen that is found in moist soils, surface waters and untreated potable water systems in tropical and subtropical regions (2, 11). Typical routes of inoculation for humans include inhalation, ingestion, and percutaneous inoculation (11, 12). *B. pseudomallei* is inherently resistant to a wide range of antibiotics including β-lactams, aminoglycosides, macrolides, and polymyxins. Effective treatment typically involves intravenous ceftazidime or meropenem for 10 to 14 days followed by oral co-trimoxazole for 3 to 6 months (11, 13–16). Previous studies have shown that antibiotic-resistant strains of *B. pseudomallei* can develop during the course of treatment and can lead to poor outcomes (11, 15, 17, 18). Even with appropriate treatment, *B. pseudomallei* infections cause significant morbidity and mortality in endemic regions.

*Burkholderia mallei* is a closely related pathogen that is transmitted to humans from solipeds (i.e. horses, mules, donkeys) and causes glanders. *B. mallei* is a genetically similar species that evolved from *B. pseudomallei* via genome reduction (19, 20). These two facultative-intracellular pathogens express similar key virulence factors including lipopolysaccharide (LPS), capsular polysaccharide (CPS), the *bsa* type III secretion system (T3SS-3) and the cluster 1 type IV secretion system (T6SS-1) (21–25). Because of this, it is conceivable that a vaccine could be designed to provide protective immunity against both melioidosis and glanders (26–30). Currently, *B. pseudomallei* and *B. mallei* are considered potential biothreat agents that are categorized as Tier 1 select agents by the U.S. Centers for Disease Control and Prevention (CDC) (31, 32). Historically, *B. mallei* was used as a biological weapon in the American Civil War and World War II (27, 33).

**Figure 1:**
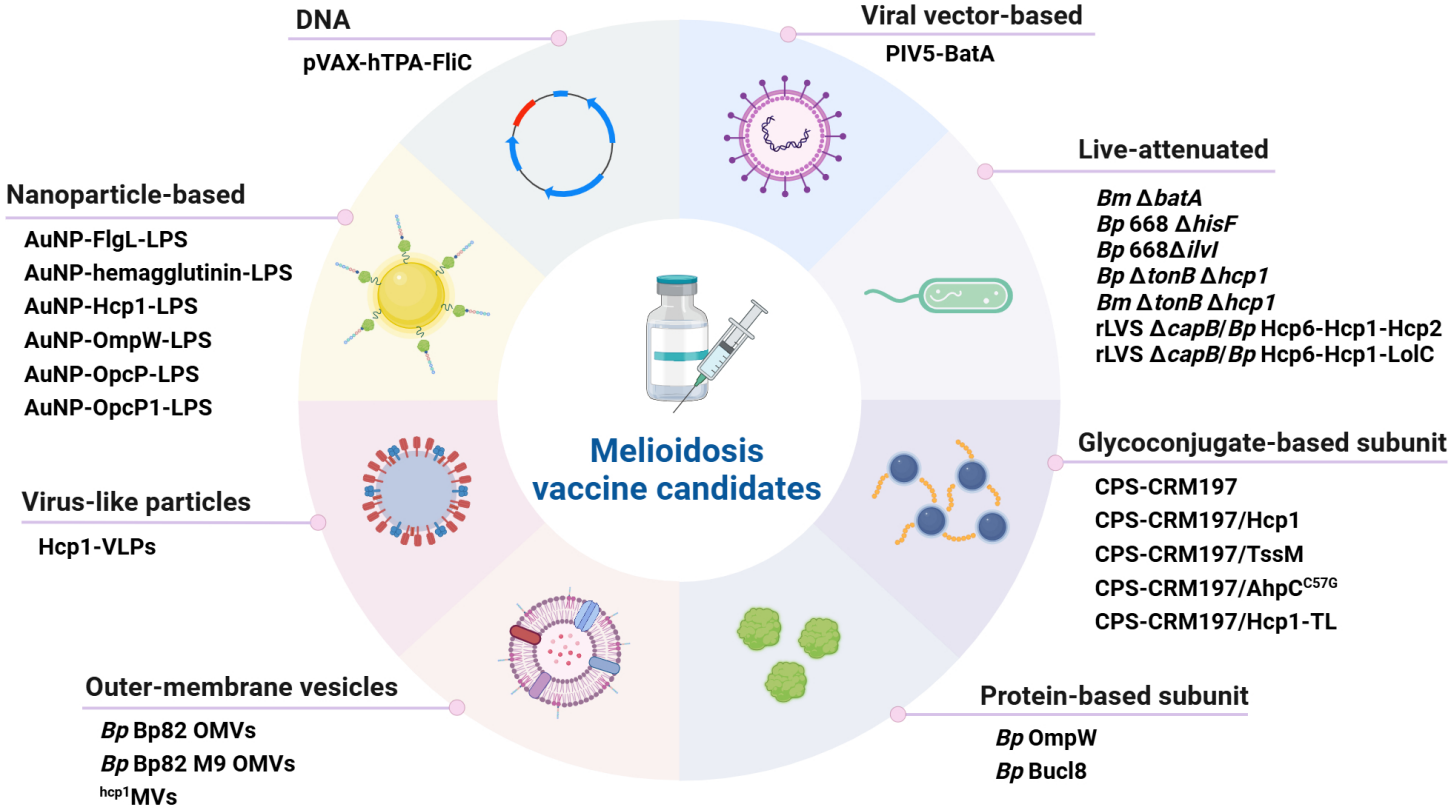
Classification of melioidosis vaccine candidates. Several different platforms have recently been used to develop a variety of promising vaccine candidates. The main types of vaccines that have been developed are i) live-attenuated, ii) glycoconjugate-based subunit, iii) protein-based subunit, iv) outer membrane vesicle, v) nanoparticle-based, vi) virus-like particles, vii) DNA and viii) viral vector-based. *Bp, B. pseudomallei*; *Bm, B. mallei*. Created in BioRender. Sengyee, S (2025). https://BioRender.com/1ypkjdc.

**Figure 2:**
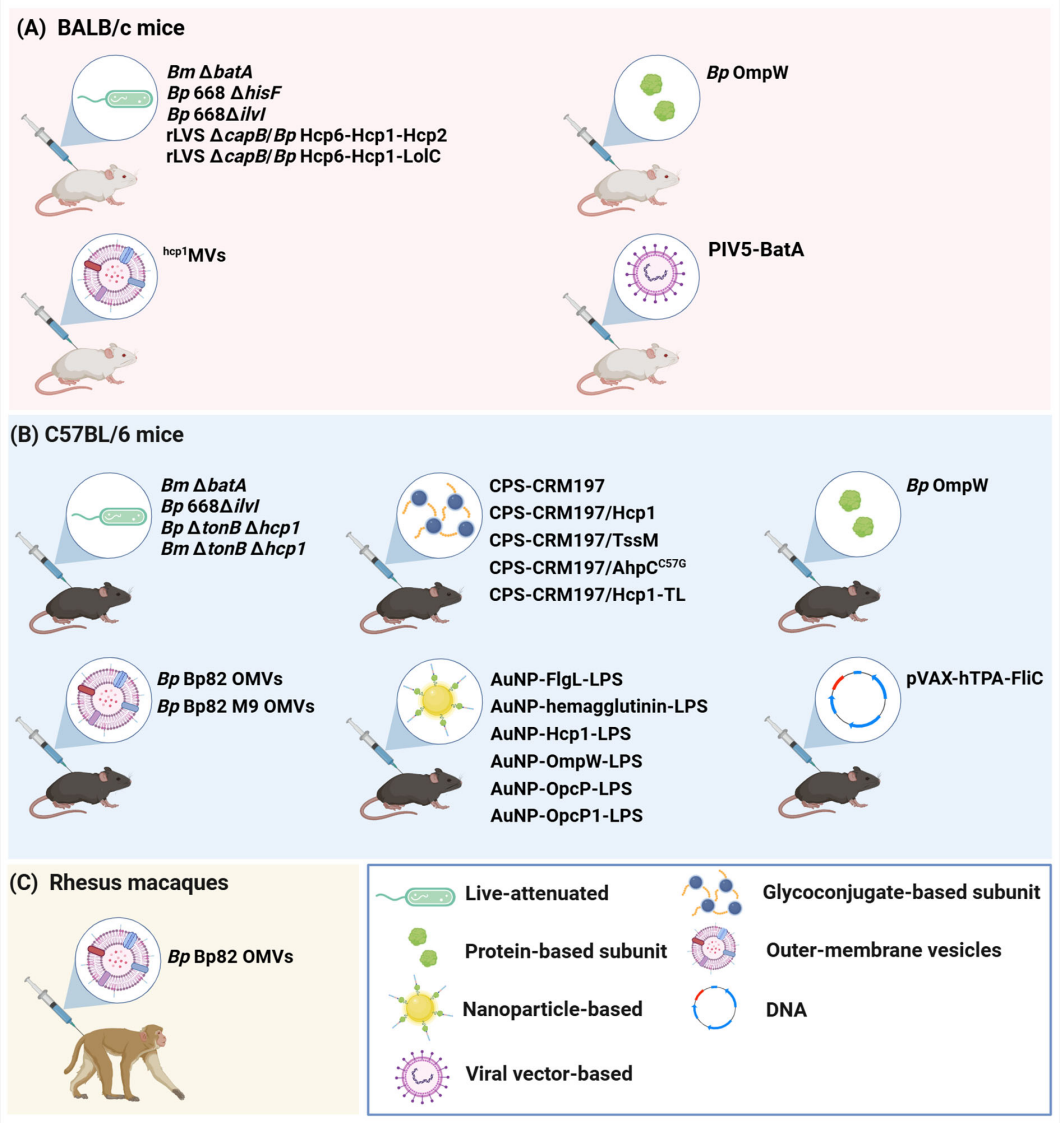
Animal models used for the development of melioidosis vaccine candidates. Three main animal models have been used to evaluate the immunogenicity and protective capacity of the melioidosis vaccine candidates discussed in this review. These are **(A)** BALB/c mice, **(B)** C57BL/6 mice and **(C)** Rhesus macaques. *Bp, B. pseudomallei*; *Bm, B. mallei*. Created in BioRender. Sengyee, S. (2025) https://BioRender.com/wdjhqlt.

An effective vaccine for immunization against melioidosis and glanders would be an important countermeasure for both public health and biodefense purposes. This review examines advances in melioidosis and glanders vaccine development over the past seven years, and focuses on work encompassing live-attenuated vaccines (LAVs), glycoconjugate-based and protein-based subunit vaccines, outer membrane vesicle (OMV) vaccines, nanoparticle-based vaccines, virus-like particle (VLP) vaccines, as well as DNA and viral vector-based vaccines (**Figure 1**). While there are currently no licensed vaccines available to protect against disease caused by these bacterial pathogens, several of the platforms discussed in this review have been shown to provide significant protection in animal models of melioidosis and/or glanders (**Table 1**; **Figure 2**).

**Table 1:** Summary of melioidosis vaccine candidates and animal challenge models.

Vaccine platform	Immunization				Challenge				Reference
	Adjuvant(s)	Route	Dose (s)	Animal model	Strain	Route	Dose CFU/animal (# of LD_50_)	Protection	
Live-Attenuated									
*Bm* Δ*batA*	–	i.t.	1	BALB/c mice	*Bp* K96243	i.t.	3 x 10^2^ (5x)	100% at day 10, 85% at day 35	(35)
				BALB/c mice	*Bp* 1026b	i.t.	2.5 x 10^4^ (5x)	71% at day 10, 67% at day 55	
				BALB/c mice	*Bm* ATCC 23344	i.t.	8 x 10^3^ (10x)	73% at day 10, 56% at day 45	
				C57BL/6 mice	*Bp* 1026b	i.t.	1.6 x 10^4^ (5x)	100% at day 35	
				C57BL/6 mice	*Bm* ATCC 23344	i.t.	9 x 10^3^ (10x)	100% at day 35	
*Bp* 668 Δ*hisF*	–	s.c.	2	BALB/c mice	*Bp* K96243	i.p.	3.1x 10^6^ (50x)	100% at day 25, 50% at day 60	(36)
*Bp* 668 Δ*ilvI*	–	s.c.	2	BALB/c mice	*Bp* K96243	i.p.	3.1x 10^6^ (50x)	100% at day 21, 50% at day 60	(36)
*Bp* 668 Δ*ilvI*	–	s.c.	2	C57BL/6 mice	*Bp* K96243	aerosol	1.35 x 10^3^ (3.4x)	60% at day 60	(37)
					*Bp* MSHR5855	aerosol	4.31 x 10^2^ (11x)	40% at day 60	
					*Bm* FMH	aerosol	7.65x 10^3^ (7.5x)	85% at day 60	
*Bp* Δ*tonB* Δ*hcp1*	–	i.n.	3	C57BL/6 mice	*Bp* K96243	aerosol	1.07 x 10^3^ (6.9x) – 1.78 x 10^3^ (11.6x)	100% at day 27	(40)
*Bm* Δ*tonB* Δ*hcp1*	–	i.n.	3	C57BL/6 mice	*Bp* K96243	aerosol	1.07x 10^3^ (6.9x) – 1.78 x 10^3^ (11.6x)	87.5% at day 27	(42)
					*Bm* ATCC 23344	aerosol	3.14 x 10^2^	100% at day 21	
					*Bm* ATCC 23344	i.n.	3.24 x 10^4^ (3x)	100% at day 21	
rLVS Δ*capB*/*Bp* Hcp6-Hcp1	–	i.d.	3	BALB/c mice	*Bp* 1026b	i.n.	2.23 x 10^3^ (5x)	88% at day 42	(44)
rLVS Δ*capB*/*Bp* Hcp6-Hcp2	–	i.d.	3	BALB/c mice	*Bp* 1026b	i.n.	2.23 x 10^3^ (5x)	88% at day 42	
rLVS Δ*capB*/*Bp* LolC-Hcp1	–	i.d.	3	BALB/c mice	*Bp* 1026b	i.n.	2.23 x 10^3^ (5x)	38% at day 42	
rLVS Δ*capB*/*Bp* LolC-Hcp2	–	i.d.	3	BALB/c mice	*Bp* 1026b	i.n.	2.23 x 10^3^ (5x)	38% at day 42	
rLVS Δ*capB*/*Bp* Hcp6-Hcp1-Hcp2	–	i.d.	3	BALB/c mice	*Bp* 1026b	i.n.	1.8 x 10^3^ (4x)	88% at day 42	
					*Bp* 1026b	i.n.	2.7 x 10^3^ (6x)	0% at day 42	
	–	i.n.	3	BALB/c mice	*Bp* 1026b	i.n.	2.7 x 10^3^ (6x)	88% at day 42	
	–	i.n.	2	BALB/c mice	*Bp* 1026b	i.n.	2.7 x 10^3^ (6x)	100% at day 42	
	–	i.n.	1	BALB/c mice	*Bp* 1026b	i.n.	2.7 x 10^3^ (6x)	86% at day 42	
rLVS Δ*capB*/*Bp* Hcp6-Hcp1-LolC	–	i.d.	3	BALB/c mice	*Bp* 1026b	i.n.	1.8 x 10^3^ (4x)	75% at day 42	
					*Bp* 1026b	i.n.	2.7 x 10^3^ (6x)	13% at day 42	
	–	i.n.	3	BALB/c mice	*Bp* 1026b	i.n.	2.7 x 10^3^ (6x)	75% at day 42	
	–	i.n.	2	BALB/c mice	*Bp* 1026b	i.n.	2.7 x 10^3^ (6x)	86% at day 42	
	–	i.n.	1	BALB/c mice	*Bp* 1026b	i.n.	2.7 x 10^3^ (6x)	88% at day 42	
Subunit									
CPS-CRM197	Alhydrogel, CpG (ODN 2006)	s.c.	3	C57BL/6 mice	*Bp* K96243	aerosol	~1.6 x 10^3^ (~10x)	67% at day 35	(48)
CPS-CRM197/Hcp1	Alhydrogel, CpG (ODN 2006)	s.c.	3	C57BL/6 mice	*Bp* K96243	aerosol	~1.6 x 10^3^ (~10x)	100% at day 35	(48)
CPS-CRM197/TssM	Alhydrogel, CpG (ODN 2006)	s.c.	3	C57BL/6 mice	*Bp* K96243	aerosol	~1.6 x 10^3^ (~10x)	80% at day 35	(48)
CPS-CRM-197/AhpC^C57G^	Alhydrogel, CpG (ODN 2006)	s.c.	3	C57BL/6 mice	*Bp* K96243	aerosol	4.04 x 10^3^ (27x) - 4.18 x10^3^ (28x)	70% at day 35	(49)
CPS-CRM197/Hcp1-TL/AhpC	Alhydrogel, CpG (ODN 2006)	s.c.	3	C57BL/6 mice	*Bp* K96243	aerosol	1.35 x 10^3^ (3.4x)	50% at day 60	(37)
CPS-CRM197/Hcp1-TL	Alhydrogel, CpG (ODN 2006)	s.c.	3	C57BL/6 mice	*Bp* K96243	aerosol	1.35 x 10^3^ (3.4x)	80% at day 60	(37)
					*Bp* MSHR5855	aerosol	4.31 x 10^2^ (11x)	35% at day 60	
					*Bm* FMH	aerosol	7.65x 10^3^ (7.5x)	80% at day 60	
*Bp* OmpW	SAS	i.p.	2	BALB/c mice	*Bp* 576	i.p.	6 x 10^5^	75% at day 21	(53)
	SAS	i.p.	2	C57BL/6 mice	*Bp* 576	i.p.	4 x 10^6^	75% at day 80	
Outer Membrane Vesicles (OMVs)									
*Bp* Bp82 OMVs	–	s.c.	2	C57BL/6 mice	*Bm* China 7	aerosol	1.25 x 10^3^ (1.4x)	80% at day 30	(89)
*Bp* Bp82 OMVs	–	s.c.	2	Rhesus macaques	*Bm* China 7	aerosol	1.6 x 10^6^ (100x)	100% at day 21	(89)
*Bp* Bp82 M9 OMVs	–	s.c.	2	C57BL/6 mice	*Bp* K96243	aerosol	1.5 x 10^3^ (8x)	100% at day 30	(91)
^hcp1^MVs	–	s.c./i.n./i.m.	3	BALB/c mice	*Bp* BPC006	i.p.	2.8 x 10^6^ (5x)	60% at day 21	(92)
^hcp1^MVs	Freund’s adjuvant	s.c./i.n./i.m.	3	BALB/c mice	*Bp* BPC006	i.p.	2.8 x10^6^ (5x)	70% at day 21	(92)
Nanoparticle-Based									
AuNP-FlgL-LPS	Alhydrogel, poly I:C	s.c.	3	C57BL/6 mice	*Bp* K96243	i.n.	1.06 x 10^5^ (3.4x)	90% at day 35	(100)
AuNP-hemagglutinin-LPS	Alhydrogel, poly I:C	s.c.	3	C57BL/6 mice	*Bp* K96243	i.n.	1.06 x 10^5^ (3.4x)	20% at day 35	(100)
AuNP-Hcp1-LPS	Alhydrogel, poly I:C	s.c.	3	C57BL/6 mice	*Bp* K96243	i.n.	1.06 x 10^5^ (3.4x)	10% at day 35	(100)
AuNP-Combo-LPS (containing FlgL, hemagglutinin, and Hcp1)	Alhydrogel, poly I:C	s.c.	3	C57BL/6 mice	*Bp* K96243	i.n.	1.06 x 10^5^ (3.4x)	100% at day 35	(100)
AuNP-OpcP-LPS	CpG (ODN 2395)	i.n.	3	C57BL/6 mice	*Bm* 23344	i.n.	2.8 x 10^4^ (2x)	100% at day 35	(101)
					*Bm* 23344	i.n.	7 x 10^5^ (50x)	80% at day 35	
AuNP-OmpW-LPS	CpG (ODN 2395)	i.n.	3	C57BL/6 mice	*Bm* 23344	i.n.	2.8 x 10^4^ (2x)	100% at day 35	(101)
					*Bm* 23344	i.n.	7 x 10^5^ (50x)	80% at day 35	
AuNP- hemagglutinin -LPS	CpG (ODN 2395)	i.n.	3	C57BL/6 mice	*Bm* 23344	i.n.	2.8 x 10^4^ (2x)	90% at day 35	(101)
					*Bm* 23344	i.n.	7 x 10^5^ (50x)	50% at day 35	
AuNP-Hcp1-LPS	CpG (ODN 2395)	i.n.	3	C57BL/6 mice	*Bm* 23344	i.n.	2.8 x 10^4^ (2x)	90% at day 35	(101)
AuNP-OpcP1-LPS	CpG (ODN 2395)	i.n.	3	C57BL/6 mice	*Bm* 23344	i.n.	2.8 x 10^4^ (2x)	70% at day 35	(101)
AuNP-FlgL-LPS	CpG (ODN 2395)	i.n.	3	C57BL/6 mice	*Bm* 23344	i.n.	2.8 x 10^4^ (2x)	40% at day 35	(101)
AuNP-Combo1-LPS (containing Hcp1, OmpW, OpcP, OpcP1, FlgL, and hemagglutinin)	CpG (ODN 2395)	i.n.	3	C57BL/6 mice	*Bm* 23344	i.n.	2.8 x 10^4^ (2x)	80% at day 35	(101)
	CpG (ODN 2395)	i.n.	3	C57BL/6 mice	*Bp* K96243	i.n.	9 x 10^4^ (6x)	0% at day 15	(102)
AuNP-Combo2-LPS (containing OmpW, OpcP, and hemagglutinin)	CpG (ODN 2395)	i.n.	3	C57BL/6 mice	*Bm* 23344	i.n.	7 x 10^5^ (50x)	100% at day 35	(101)
AuNP-OpcP-LPS	CpG (ODN 2395)	i.n.	3	C57BL/6 mice	*Bp* K96243	i.n.	7.5 x 10^4^ (5x)	90% at day 35	(102)
					*Bp* K96243	i.n.	9 x 10^4^ (6x)	90% at day 35	
AuNP-OpcP1-LPS	CpG (ODN 2395)	i.n.	3	C57BL/6 mice	*Bp* K96243	i.n.	7.5 x 10^4^ (5x)	80% at day 35	(102)
					*Bp* K96243	i.n.	9 x 10^4^ (6x)	30% at day 35	
AuNP-Combo2-LPS (containing OpcP and OpcP1)	CpG (ODN 2395)	i.n.	3	C57BL/6 mice	*Bp* K96243	i.n.	7.5 x 10^4^ (5x)	100% at day 35	(102)
DNA									
pVAX-hTPA-FliC	Polyethylenimine	i.n.	1	C57BL/6 mice	*Bp* 1026b	i.n.	5 x 10^2^ (1x)	53% at day 14	(110)
Virus Vector-Based									
PIV5-BatA	–	i.n.	1	BALB/c mice	*Bp* K96243	aerosol	3 x 10^2^ (5x)	80% at day 10 and 60% at day 35	(114)
					*Bm* ATCC 23344	aerosol	8 x 10^3^ (10x)	84% at day 10 and 74% at day 40	

*Bp*, *B. pseudomallei*; *Bm*, *B. mallei*; i.t., intratracheal; s.c., subcutaneous; i.p., intraperitoneal; i.n., intranasal; i.d., intradermal; i.m., intramuscular.

## 2 Vaccine platforms

### 2.1 Live-attenuated vaccines

LAV strains designed to provide protection against melioidosis and/or glanders have been developed and evaluated in both BALB/c and C57BL/6 mouse models (**Table 1**). While some LAV strains have been shown to induce protective immunity against *B. pseudomallei*, these types of vaccines have associated risks including the possibilities of reversion to wild-type virulence and development of latent infections (34). Recently, *B. mallei* Δ*batA*, which harbors a mutation in the autotransporter protein BatA, was used to immunize mice against lethal intratracheal challenges with *B. mallei* and *B. pseudomallei* (35). Immunization of BALB/c and C57BL/6 mice with 10^4^ CFU of this LAV strain stimulated robust antibody responses and resulted in 56-100% and 67-100% survival against challenges with *B. mallei* and *B. pseudomallei*, respectively (**Table 1**) (35). Furthermore, analysis of humoral immune responses from BALB/c mice immunized with Δ*batA* demonstrated that robust *B. mallei*-specific IgG titers were generated with a strong Th1-bias (as evidenced by high IgG2a/IgG1 ratios) and that the serum enhanced uptake of opsonized bacteria as well as promoted effective intracellular killing by macrophages (35). In addition, passive transfer of the immune serum to mice provided equivalent levels of protection to Δ*batA* immunized mice when they were challenged intratracheally with *B. mallei* or *B. pseudomallei* (35).

Several different single gene auxotrophs constructed in *B. pseudomallei* MSHR668 have been evaluated as LAVs in BALB/c mice with the most effective strains being *B. pseudomallei* 668 Δ*hisF* and 668 Δ*ilvI* (36). Subcutaneous immunization of BALB/c mice with two doses of *B. pseudomallei* 668 Δ*hisF* or 668 Δ*ilvI* demonstrated similar levels of protection against intraperitoneal challenges with a 50-fold median lethal dose (MLD_50_) of *B. pseudomallei* K96243, with survival rates of 100% at day 25 or 21, respectively, and 50% at day 60 (**Table 1**) (36). The serum levels of *B. pseudomallei*-specific IgG were similar in mice immunized with *B. pseudomallei* 668 Δ*hisF* and 668 Δ*ilvI* (36). Upon re-stimulation, splenocytes obtained from mice immunized with *B. pseudomallei* 668 Δ*hisF* or 668 Δ*ilvI* displayed significantly increased IFN-γ cytokine responses compared to the phosphate buffered saline control group, suggesting that cellular immune responses contribute to protection against *B. pseudomallei* infection (36).

Additionally, the *B. pseudomallei* 668 Δ*ilvI* LAV was evaluated in C57BL/6 mice, and results revealed that subcutaneous immunizations with this strain conferred survival rates of 40-60% and 85% at day 60 against lethal aerosol challenges of *B. pseudomallei* and *B. mallei*, respectively (**Table 1**) (37). IgG responses were measured against killed whole-cells and purified *B. pseudomallei* O-polysaccharide (OPS) and shown to correlate with protection in *B. pseudomallei* 668 Δ*ilvI-*immunized C57BL/6 mice. Analysis of cytokine profiles of lung homogenates obtained post-challenge with *B. pseudomallei* K96243 revealed that the levels of IFN-γ and IL-22 had increased significantly suggesting that these cytokines correlated with protective immunity in the surviving mice (37). Recently, a combination of *B. pseudomallei* 668 Δ*ilvI* vaccination and co-trimoxazole treatment delivered every 12 hours for either 7 or 21 days demonstrated improved protection in C57BL/6 mice against an inhalational challenge with *B. pseudomallei* K96243 compared to immunized mice with no post-exposure antibiotic co-treatment. This combined approach provided 80-100% survival for up to 86 days post challenge (38).

Safety concerns associated with LAV strains include tolerance induction, autoimmune exacerbation, and reversion to wild type virulence. Because of this, introducing attenuating mutations at multiple sites is preferred to reduce the chances of reversion to virulent phenotypes (39). To address this concern, strains harboring mutations in the *tonB* and *hcp1* genes were constructed in both *B. pseudomallei* K96243 and *B. mallei* ATCC 23344. These Δ*tonB* Δ*hcp1* double mutants were deficient in iron acquisition, intracellular spread, and ability to stimulate multinucleated giant cell formation (40–43). Immunization of C57BL/6 mice with the *B. pseudomallei* Δ*tonB* Δ*hcp1* strain stimulated strong Th1-biased humoral immune responses (IgG2a > IgG1) when serum was titered against irradiated *B. pseudomallei* K96243. Additionally, robust IFN-γ, TNF-α and IL-17A cytokine production was observed in cell supernatants following the re-stimulation of splenocytes with heat-killed *B. pseudomallei* K96243 whole cell lysates (40). Upon a lethal inhalational challenge with *B. pseudomallei*, 100% of the mice immunized with *B. pseudomallei* Δ*tonB* Δ*hcp1* survived until day 27, exhibited low bacterial loads (less than 20 CFU/organ) and minimal pathological changes in lungs, livers, and spleens (**Table 1**) (40). When mice depleted of CD4^+^ or CD8^+^ T cells were immunized with *B. pseudomallei* Δ*tonB* Δ*hcp1* and then challenged with *B. pseudomallei* K96243 results demonstrated that the absence of these T cells did not significantly affect the levels of survival, suggesting that protective immunity against *B. pseudomallei* primarily correlated with humoral immune responses in this study (40).

Recently, the cross-protective properties of *B. mallei* Δ*tonB* Δ*hcp1* have been examined in mouse models of both glanders and melioidosis (42, 43). *B. mallei* Δ*tonB* Δ*hcp1* provided C57BL/6 mice with 100% protection at day 21 against both intranasal and inhalational challenges of *B. mallei* ATCC 23344, and 87.5% protection at day 27 following an inhalational challenge with *B. pseudomallei* K96243 (**Table 1**) (42). The surviving mice demonstrated significant reductions in bacterial burdens in the lungs, livers, and spleens with 87.5% and 50% sterilizing immunity in the intranasal and inhalational challenge experiments, respectively (42). Immunization with *B. mallei* Δ*tonB* Δ*hcp1* stimulated high levels of *B. mallei*-specific IgG, IgG1, and IgG2a in serum as well as robust IFN-γ and IL-17A cytokine responses in cell supernatants following re-stimulation of splenocytes with heat-killed *B. mallei* ATCC 23344 or *B. pseudomallei* K96243 whole cell lysates (42). Consistent with previous findings, depletion of CD4^+^ or CD8^+^ T cells showed no difference in levels of protection or bacterial burdens in immunized mice. These results supported that humoral immune responses play a major role in the protective capacity of the Δ*tonB* Δ*hcp1* LAV strains (40, 42).

Khakhum et al. also evaluated the immune correlates of protection following the immunization of C57BL/6 mice with *B. pseudomallei* Δ*tonB* Δ*hcp1* and *B. mallei* Δ*tonB* Δ*hcp1*. Their results confirmed that both LAV strains elicited strong *B. pseudomallei*-specific serum IgM, IgG2b, and IgG2c responses that promoted bacterial uptake and enhanced bacterial killing by macrophages (43). However, passive transfer of serum from mice immunized with the Δ*tonB* Δ*hcp1* LAV strains to naïve mice did not provide protection against inhalational challenges with *B. pseudomallei* K96243 (43). Interestingly, the Δ*tonB* Δ*hcp1* LAV strains stimulated robust mucosal immune responses in the lungs, particularly IgA as well as Th1- and Th17-like CD4^+^ T cell responses. Histological analysis of lung tissues from immunized mice challenged with *B. pseudomallei* revealed only mild to moderate lung inflammation, suggesting that controlled immune activation stimulated protective immunity (43).

A more recent study by Tullius et al., used derivatives of the *Francisella tularensis* Live Vaccine Strain (LVS) Δ*capB* that were engineered to express *B. pseudomallei* antigens as novel vaccine candidates (44). LVS, derived from *F. tularensis subsp. holarctica*, is a less virulent subspecies of *F. tularensis* that has been previously used to construct vaccine candidates for tularemia, anthrax, plague, and COVID-19 (44). LVS Δ*capB*, a mutant lacking a putative capsule synthesis gene, expressing the *B. pseudomallei* T6SS proteins Hcp1, Hcp2, or Hcp6, or the membrane protein LolC were constructed in different combinations and evaluated for their immunogenicity and protective capacity in BALB/c mice. LVS Δ*capB* alone or expressing two, three or four *B. pseudomallei* proteins (rLVS Δ*capB*/*Bp* proteins) were used to immunize mice via either an intranasal or an intradermal route and then challenged intranasally with 5 LD_50_ of *B. pseudomallei* 1026b. The mice that were immunized intradermally with rLVS Δ*capB*/*Bp*-Hcp6-Hcp1 or rLVS Δ*capB*/*Bp*-Hcp6-Hcp2 exhibited 88% survival at day 42 post-challenge, while groups that were immunized with rLVS Δ*capB*/*Bp*-LolC-Hcp1 or rLVS Δ*capB*/*Bp*-LolC-Hcp2 exhibited only 38% survival over the same timeframe (**Table 1**). The use of the rLVS Δ*capB*/*Bp*-Hcp6-Hcp1 and rLVS Δ*capB*/*Bp*-Hcp6-Hcp2 strains resulted in high levels of sterilizing immunity with 75% and 50% of the mice surviving until day 42, respectively (44).

Since rLVS Δ*capB*/*Bp*-Hcp6-Hcp1 showed promising results with high survival rates and sterilizing immunity, rLVS Δ*capB*/*Bp* vaccine candidates expressing three antigens (Hcp6-Hcp1-Hcp2 or Hcp6-Hcp1-LolC) were constructed. Mice immunized intradermally with rLVS Δ*capB/Bp*-Hcp6-Hcp1-Hcp2 or rLVS Δ*capB/Bp*-Hcp6-Hcp1-LolC and then challenged with 4 LD_50_ of *B. pseudomallei* 1026b intranasally yielded survival rates of 88% and 75%, respectively (**Table 1**). Additionally, intranasal administration of rLVS Δ*capB/Bp*-Hcp6-Hcp1-Hcp2 or rLVS Δ*capB/Bp*-Hcp6-Hcp1-LolC provided 75-100% protection against intranasal challenges with 6 LD_50_ of *B. pseudomallei* 1026b (**Table 1**). Notably, intranasal delivery of the various rLVS Δ*capB*/*Bp* vaccine candidates proved to be superior to intradermal delivery and provided robust protection against both low and high challenge doses (**Table 1**) (44). As observed in previous studies, humoral immunity appeared to dominate the *B. pseudomallei* antigen-specific immune responses, as all groups immunized with rLVS Δ*capB*/*Bp* strains generated strong serum IgG titers against Hcp1, Hcp6, and LolC. However, no significant increases in antigen-specific T-cell responses were observed (44). While these findings highlight the promising nature of rLVS Δ*capB*/*Bp* vaccine candidates, particularly in inducing robust humoral immunity, the lack of significant T-cell responses suggests that further refinement to enhance cellular immunity may be necessary for optimizing protection against melioidosis.

### 2.2 Subunit vaccines

Subunit vaccines are composed of one or more purified antigens that induce protective immune responses and are typically formulated with immune-stimulating adjuvants. Adjuvants are used to enhance both humoral and cellular immune responses against the antigens by activating innate immune receptors, promoting antigen uptake and processing/presentation, and stimulating Th1-, Th2- and/or Th17-like responses. For instance, Alhydrogel is known to promote potent Th2-like responses, while monophosphoryl lipid A and CpG oligodeoxynucleotides (CpG) are associated with development of strong Th1-like responses (45–47). Subunit vaccines have several advantages over the use of LAVs including that they are safe, antigenically defined, and pose minimal risk to immunocompromised individuals following immunization. Additionally, they allow for the selection of conserved protective antigens that are capable of eliciting robust immune responses against multiple species or strains within a species (48–51). Limitations of subunit vaccines compared to other platforms, however, are their need for adjuvants and the requirement for multiple doses to achieve optimal protection. Furthermore, production of antigens for these types of vaccines can be technically challenging and costly.

Some of the most recently developed melioidosis subunit vaccines consist of glycoconjugate-based and protein-based formulations (48–54). Glycoconjugate-based subunit vaccines are produced via the covalent linkage of bacterial polysaccharides to carrier proteins to facilitate linked recognition and promote the development of T cell-dependent type immune responses against the polysaccharide component of these hybrid immunogens (48–52). A non-toxic mutant of diphtheria toxin, cross-reacting material 197 (CRM197) is a commonly used protein carrier for glycoconjugate vaccines. CRM197 is used in *Haemophilus influenza* type b, *Streptococcus pneumoniae* and *Neisseria meningitidis* conjugate vaccines, as well as in the *Burkholderia* polysaccharide-based glycoconjugate vaccine candidates discussed here (55, 56).

Two important and highly conserved polysaccharide antigens expressed by both *B. pseudomallei* and *B. mallei* are the virulence associated 6-deoxyheptan CPS (21, 25, 50, 57–60) and the O-polysaccharide (OPS) moieties of LPS (25, 26, 58, 61, 62). Previous studies have shown that immunization with purified *Burkholderia* CPS and/or LPS provides high levels of protection in mouse models of melioidosis (26, 58, 62). Several studies have also found that CPS- and OPS-specific monoclonal antibodies (mAbs) provide protection against intraperitoneal *B. pseudomallei* challenges in rats and/or BALB/c mice (30, 63, 64). Additionally, it has been observed that anti-OPS antibody responses were significantly higher in melioidosis survivors in northeastern Thailand compared to individuals who succumbed to infection (65).

Since *B. pseudomallei* and *B. mallei* express structurally similar OPS moieties, these antigens are considered as potential candidates for use in the development of glycoconjugate vaccines that could provide protection against both melioidosis and glanders (66). Tamigney Kenfack et al. demonstrated that OPS-specific mAbs exhibited strong interactions with the 6-deoxytalose residue of the 3-O-methylated terminal disaccharides of *B. mallei* or *B. pseudomallei* OPS (67). In their study, they constructed synthetic oligosaccharide conjugates (SOC-6 and SOC-7) which represented the terminal disaccharides of *B. mallei* or *B. pseudomallei* OPS linked to CRM197, and then evaluated their immunogenicity in BALB/c mice. These synthetic OPS-based glycoconjugates stimulated high levels of antigen-specific IgG, with SOC-6 eliciting higher titers than SOC-7 (67). Enzyme-linked immunosorbent assay (ELISA) results showed that culture-confirmed Thai melioidosis patient samples were also reactive to these synthetic OPS-based glycoconjugates, suggesting that synthetic or native OPS may potentially be useful as a vaccine antigen (67).

CPS is also an attractive antigen for glycoconjugate vaccine development since it is highly conserved in virulent isolates of *B. pseudomallei* and *B. mallei* (21, 25, 50, 57–60). When conjugated to the carrier protein CRM197 to form the glycoconjugate CPS-CRM197, T cell dependent-like responses are raised against the polysaccharide component of the molecule, resulting in high-titer CPS-specific antibody responses in C57BL/6 mice (48, 49). In addition to polysaccharides, *B. pseudomallei* and *B. mallei* also express several conserved protein antigens including the T6SS-1 associated hemolysin coregulated protein 1 (Hcp1) and the deubitiquinase (TssM), which have been shown to be immunogenic in animal models and correlate with survival in melioidosis patients from Thailand (68, 69).

A recent study focused on the development of CPS-based glycoconjugate subunit vaccine candidates assessed the immunogenicity and protective capacity of CPS-CRM197 when combined with either Hcp1 or TssM. Immunization of C57BL/6 mice with CPS-CRM197, CPS-CRM197 plus Hcp1, or CPS-CRM197 plus TssM, all formulated with Alhydrogel and CpG (ODN 2006) resulted in 67%, 100%, and 80% protection, respectively, at 35 days following an acute inhalational challenge with *B. pseudomallei* K96243 (**Table 1**) (48). Notably, 70% of the mice immunized with CPS-CRM197 plus Hcp1 formulation that survived the duration of the experiment had no culturable bacteria in lungs, livers, or splenic tissues. All three test groups produced high titer IgM and IgG responses against CPS. Mice immunized with CPS-CRM197 plus Hcp1 or CPS-CRM197 plus TssM stimulated high-titer IgM and IgG against their respective recombinant *Burkholderia* proteins. Further analysis of immune serum showed that antibody responses to all antigens were Th1/Th2 balanced based on the IgG2b/IgG1 ratios. Robust IFN-γ-secreting T responses were also observed when splenocytes were re-stimulated with either Hcp1 or TssM (48).

Another highly immunogenic protein that has been identified as a potential vaccine candidate is alkyl hydroperoxide reductase subunit C (AhpC), which is involved in protecting cells from oxidative damage (70). Previous studies have shown that enhanced T-cell responses to AhpC correlate with survival in melioidosis patients, highlighting its potential as a protective antigen (71). In recent studies, CPS-CRM197 plus AhpC harboring an active site mutation (AhpC^C57G^) formulated with Alhydrogel and CpG (ODN 2006) was used to immunize C57BL/6 mice prior to an inhalational challenge with *B. pseudomallei* K96243 (49). This formulation elicited high levels of protection, with 70% of immunized mice surviving to day 35 (**Table 1**) (49). Survival rates were significantly higher than the adjuvant-only control mice but were lower than the levels of protection observed in prior studies using CPS-CRM197 plus Hcp1 or TssM (48). CPS-CRM197 plus AhpC^C57G^ immunized mice produced high titer CPS- and AhpC^C57G^-specific serum IgG responses, and robust IFN-γ-, IL-5-, and IL-17-secreting T cell responses following the re-stimulation of splenocytes against AhpC^C57G^ (49).

More recently, CPS-CRM197 was used to immunize C57BL/6 mice along with Hcp1-TL and AhpC^C57G^, or Hcp1-TL alone, both formulated with Alhydrogel and CpG (ODN 2006) (37). Mice in the CPS-CRM197 plus Hcp1-TL/AhpC^C57G^ and CPS-CRM197 plus Hcp1-TL groups were challenged with *B. pseudomallei* K96243 via an inhalational route, and resulted in survival rates of 50% and 80% by day 60, respectively (**Table 1**) (37). In a second study, mice immunized with CPS-CRM197 plus Hcp1-TL were challenged via an inhalational route with *B. mallei* FMH and *B. pseudomallei* MSHR5855, and showed survival rates of 80% and 35% by day 60, respectively (**Table 1**) (37). In addition, the CPS-CRM197 plus Hcp1-TL formulation was shown to produce similar levels of protection, cellular and humoral immune responses, and sterilizing immunity when compared to the LAV strain 668 Δ*ilvI* (37).

Extending upon these studies, novel intervention strategies that layer vaccination and post-exposure antibiotic treatment have been conducted with CPS-CRM197-based subunit vaccine candidates. When C57BL/6 mice were immunized with CPS-CRM197 plus Hcp1-TL/AhpC^C57G^ or CPS-CRM197 plus Hcp1-TL, both formulated with Alhydrogel and CpG (ODN 2006), in combination with co-trimoxazole treatment and then challenged with *B. pseudomallei* K96243 via an inhalational route, 90-100% of mice survived to day 86 (37, 38). CPS-CRM197 plus Hcp1 was also evaluated in combination with the fluoroquinolone antibiotic, finafloxacin, against inhalational challenges of *B. pseudomallei* K96243 in BALB/c mice (72). In this study, mice were immunized subcutaneously with CPS-CRM197 plus Hcp1 formulated with Alhydrogel alone or CPS-CRM197 plus Hcp1 formulated with Alhydrogel and CpG (ODN 2006) and finafloxacin treatment was initiated at 36 or 48 h post-challenge. Notably, the formulation resulted in a synergistic effect only when CpG (ODN 2006) was included and when finafloxacin treatment was started at 48 h post-challenge. Mice that were immunized with CPS-CRM197 plus Hcp1 formulated with Alhydrogel and CpG (ODN 2006) and treated with finafloxacin exhibited 80% survival up to 35 days post challenge with *B. pseudomallei* K96243. In contrast, groups that were immunized with CPS-CRM197 plus Hcp1 formulated with Alhydrogel alone and then treated with finafloxacin showed only 40% survival (72).

Several *B. pseudomallei* protein antigens including LolC, PotF, OppA, and various outer membrane proteins (e.g., Omp3, Omp7, Omp85 and OmpW) have been identified and evaluated as potential candidates for use in protein-based subunit vaccines (53, 73–75). Casey et al. assessed the protective efficacy of OmpW formulated with the Sigma-adjuvant system (SAS), which is composed of monophosphoryl lipid A (TLR-4 ligand) and trehalose dicorynomycolate (a C-type lectin mincle receptor ligand), in both BALB/c and C57BL/6 mouse models (53). Intraperitoneal immunization of mice with SAS-adjuvanted OmpW followed by lethal intraperitoneal challenges of *B. pseudomallei* 576 resulted in 75% survival in both BALB/c mice (day 21) and C57BL/6 mice (day 80) (**Table 1**) (53). Immunization with SAS-adjuvanted OmpW elicited strong serum antibody responses, along with IFN-γ-secreting CD4^+^, CD8^+^, natural killer, and natural killer T cell responses against OmpW in non-insulin-resistant C57BL/6J and insulin-resistant C57BL/6J mouse models of Type 2 diabetes (53, 76). While SAS is a highly effective adjuvant, it has not been approved for human use.

Tomás-Cortázar et al. proposed CAF01 as a promising adjuvant, since it has a proven human safety profile and has demonstrated efficacy against various intracellular pathogens, such as tuberculosis and malaria (54). In their study, C57BL/6J mice were immunized subcutaneously with CAF01-adjuvanted OmpW or with CAF01 alone. Quantitation of serum antibody levels indicated balanced Th1/Th2 responses based on the IgG2a/IgG1 ratios. Upon re-stimulation with *B. pseudomallei* OmpW, splenocytes obtained from mice immunized with CAF01-adjuvanted OmpW demonstrated robust OmpW-specific Th1 (IFN-γ), Th2 (IL-4), and Th17 (IL-17) responses (54). CAF01-adjuvanted OmpW was found to stimulate equivalent or superior immune responses when compared to OmpW combined with the SAS adjuvant, making it a promising candidate for future studies (54, 76). These studies suggested that *B. pseudomallei* OmpW adjuvanted with CAF01 has the potential to be an effective vaccine candidate for melioidosis. However, the protective capacity of this vaccine formulation still needs to be evaluated in animal challenge experiments to determine its protective efficacy as a melioidosis vaccine candidate.

Another candidate antigen that has been investigated as a potential protein-based subunit vaccine candidate is the outer membrane protein *Burkholderia* collagen-like 8 (Bucl8). Bucl8 is composed of two main components (i) a periplasmic α- and outer membrane β-barrels (ii) an extended extracellular portion composed of a collagen (CL) domain and a non-collagenous carboxyl terminal (Ct) region (77). As part of a novel tetrapartite efflux pump, Bucl8 plays a crucial role in fusaric acid resistance, fibrinogen binding, and optimal growth, making it an attractive target for vaccine development (77). Additionally, homology modelling has identified extracellular loops 1 and 2 (L1 and L2) on the β-barrel, and the extended extracellular CL-Ct portion as promising vaccine antigens (77, 78). In studies with CD-1 mice, subcutaneous immunization with recombinant proteins Bucl8-CL/Ct or synthetic peptide L1- or L2-CRM197 conjugates promoted strong Th2 (IgG1) antibody responses against the corresponding proteins or peptides (78). Interestingly, peptide-conjugate L1 elicited significantly higher antibody titers compared to L2, suggesting differential immunogenicity between the two loops (78). However, this subcutaneous immunization failed to provide protection against an intranasal challenge with *B. thailandensis* strain E264, suggesting that the lack of mucosal immunity may have contributed to this failure (78, 79). To enhance mucosal immunity, intranasal immunization with L1-CRM197 formulated with fluorinated cyclic diguanosine monophosphate (FCDG) was tested (79). This approach also failed to protect CD-1 mice against an intranasal challenge of 8 × 10^5^ CFU of *B. thailandensis* strain E264 (79). While Bucl8 showed promise as a subunit vaccine candidate further optimization in these studies additional testing using a *B. pseudomallei* challenge is necessary to further evaluate its potential as a subunit vaccine candidate for melioidosis.

### 2.3 Outer membrane vesicle vaccines

OMVs are non-infectious vesicles that are constitutively secreted by Gram-negative bacteria (80). They are composed of numerous virulence factors and Toll-like receptor agonists that aid in the activation of immune cells and are, thus, self-adjuvating (81, 82). The use of OMVs as a vaccine platform is desirable as it is inherently safer than LAVs due to the absence of self-replicative capacity. Previously, OMV vaccines have provided protection and elicited robust immune responses against *Klebsiella pneumoniae, Neisseria meningitidis*, and *Bordetella pertussis* (83–85). Recent work has shown that OMVs derived from *B. pseudomallei* provided protection against lethal inhalational challenges of *B. pseudomallei* and *B. mallei* in C57BL/6 mice and NHP models of infection (86–88). OMVs derived from *B. pseudomallei* 1026b provided BALB/c mice with 67% and 60% protection in lethal sepsis and pulmonary infection models, respectively, but did not result in sterilizing immunity (86, 87).

The same research group recently demonstrated that OMVs derived from the select agent-excluded strain *B. pseudomallei* Bp82, a Δ*purM* mutant of strain 1026b, provided cross-protection against inhalational challenges of *B. mallei* in both C57BL/6 mice and NHPs (**Table 1**) (89, 90). Mice immunized with the OMV vaccine generated high titer OMV-specific and *B. mallei*-specific serum IgG responses as well as robust *B. mallei-*specific Th1/Th17 CD4^+^ and CD8^+^ T cell responses (89). C57BL/6 mice immunized with *B. pseudomallei* Bp82-derived OMVs displayed humoral and cellular immune responses that were comparable to mice immunized with *B. pseudomallei* Bp82 when used as a LAV strain. In challenge experiments, immunization with Bp82 derived OMVs resulted in 80% of mice surviving to day 30. Rhesus macaques immunized with OMVs and then challenged with *B. mallei* displayed sub-clinical infections with pulmonary lesions and mild bronchopneumonia, with 100% of the animals surviving up to the 30 day study endpoint (**Table 1**) (89). High levels of *B. mallei-*specific and OMV-specific serum IgG were also observed in immunized Rhesus macaques when compared to saline only controls. There were no detectable differences in cellular immune responses between OMV- and control animals (89). This OMV platform is comparable in immunogenicity and protective capacity to *B. pseudomallei* Bp82 when used as a LAV strain and displayed cross-protection to *B. mallei*, but similar to previous work failed to produce sterilizing immunity (86, 87, 89).

More recently, the OMV platform was improved upon by generating OMVs from *B. pseudomallei* Bp82 grown in M9 minimal media (M9 OMV) (91). This nutrient-limiting media mimics the intracellular environment of a macrophage, enriching OMVs with intracellular-stage proteins associated with virulence and key immune targets that are predicted to be important for providing sterilizing immunity. One immunogenic protein found to be enriched in M9 OMVs as compared to earlier OMVs is Hcp1, a component of T6SS-1 (68, 69, 91). Following immunization with *B. pseudomallei* Bp82 LAV or M9 OMVs derived from *B. pseudomallei* Bp82, C57BL/6 mice were challenged with *B. pseudomallei* K96243 via an inhalational route (91). The M9 OMV vaccine conferred 100% protection at day 30, and spleens collected at the study endpoint yielded no culturable bacteria (**Table 1**) (91). M9 OMV immunized mice produced significantly higher IgG titers to OMVs and whole inactivated bacteria than mice immunized with *B. pseudomallei* Bp82 LAV. Similar trends were observed for cellular immune responses in that IFN-γ- and IL-17-secreting CD4^+^ T cells and IFN-γ-secreting CD8^+^ T cells were higher in M9 OMV immunized mice than *B. pseudomallei* Bp82 LAV immunized mice (91). These results demonstrate that M9 OMVs not only offer improved immunogenicity and protection comparable to LAVs but also represent a promising vaccine candidate that may be capable of achieving sterilizing immunity.

Previous work has demonstrated that Hcp1 elicits strong IFN-γ-secreting T cell responses that correlate with survival in melioidosis patients (68). Building upon this, a recent study engineered a *Staphylococcus aureus* strain, RN4220-Δ*agr*/*pdhB-hcp1*, to produce Hcp1-loaded OMVs (92). This involved the construction of an in-frame fusion of the *hcp1* gene from *B. pseudomallei* BPC006 with the gene encoding a major vesicular component in *S. aureus* RN4220-Δ*agr*. To generate *B. pseudomallei* Hcp1-loaded membrane vesicles (^hcp1^MVs), RN4220-Δ*agr/pdhB-hcp1* was cultured and subjected to a series of centrifugation and filtration steps. BALB/c mice were immunized with three doses of ^hcp1^MVs alone or ^hcp1^MVs formulated with Freund’s adjuvant and then challenged with *B. pseudomallei* BPC006 via the intraperitoneal route. ^hcp1^MVs- and ^hcp1^MVs/Freund’s adjuvant-immunized mice displayed 60% and 70% survival over 21 days, respectively (**Table 1**) (92). Following immunization, mice that received ^hcp1^MVs/Freund’s adjuvant displayed the highest titer IgG responses to Hcp1 (92). This study suggests that OMVs loaded with *B. pseudomallei* antigens may be potential melioidosis vaccine candidates when combined with an appropriate adjuvant.

### 2.4 Nanoparticle-based vaccines

Gold nanoparticles (AuNPs) are promising candidates for various biological applications due to their unique physical properties, ease of synthesis, capacity for bioconjugation with protein or polysaccharide antigens as well as their utility as vaccine delivery systems (93, 94). AuNPs covalently coupled to one of three different proteins (Hcp1, Hc fragment of tetanus toxin, or flagellin) and LPS purified from the lowly-pathogenic species, *B. thailandensis*, have been evaluated in BALB/c mice for protection against glanders (95). These vaccine candidates induced LPS-specific IgG responses and provided 60-90% survival at day 35 following a lethal inhalational challenge with *B. mallei* strain ATCC 23344 (95). When evaluated in Rhesus macaques, an AuNP glycoconjugate vaccine composed of *B. thailandensis* LPS conjugated to flagellin formulated with Alhydrogel generated high titer LPS- and protein-specific IgG responses, however, 50% survival was observed against a *B. mallei* challenge (96).

Recently, a reverse vaccinology approach was employed to identify novel protein candidates that are conserved in both *B. pseudomallei* and *B. mallei* for potential use in AuNP glycoconjugate-based vaccines. Candidates were selected based on predicted antigenicity and validated by confirming their reactivity with melioidosis sera from humans and mice (97–102). Three promising candidates (FlgL, hemagglutinin, and Hcp1) were identified and individually conjugated to an AuNP-glycoconjugate platform along with *B. thailandensis* LPS (100). The AuNP-glycoconjugate formulations were used alone or in combination with the three proteins (AuNP-combo-LPS) to subcutaneously immunize C57BL/6 mice. When intranasally challenged with 3.4 LD_50_ of *B. pseudomallei* K96243, mice receiving AuNP-FlgL-LPS and AuNP-combo-LPS demonstrated the highest levels of protection with 90% and 100% survival at day 35, respectively (**Table 1**). Groups receiving AuNP-hemagglutinin-LPS and AuNP-Hcp1-LPS exhibited 20% and 10% survival, at day 35, respectively (**Table 1**) (100). Surviving mice in all groups demonstrated a significant reduction of bacterial loads in lungs compared to the adjuvant-only control group (100).

Additional AuNP-glycoconjugate vaccine candidates have been developed by incorporating predicted immunogenic proteins such as OmpW, OpcP, and OpcP1 along with previously identified antigens (101, 102). AuNP-protein-LPS candidates, comprised of different proteins coupled to AuNPs and LPS were tested in mouse models of glanders (101). C57BL/6 mice that received AuNP-OpcP-LPS, AuNP-OmpW-LPS, AuNP-hemagglutinin-LPS or AuNP-Hcp1-LPS demonstrated high level protection (90-100% survival) at day 35 following an intranasal challenge with 2 LD_50_ of *B. mallei* ATCC 23344 (**Table 1**). Since the protection afforded by AuNP-OpcP-LPS, AuNP-OmpW-LPS or AuNP-hemagglutinin-LPS was significantly higher than the adjuvant control group, these formulations were further evaluated using a higher challenge dose (50 LD_50_). An AuNP-glycoconjugate vaccine containing a combination of three proteins (OpcP, OmpW, and hemagglutinin) and LPS resulted in 100% survival at 35 days following an intranasal challenge with 50 LD_50_ of *B. mallei* ATCC 23344, while AuNP-OpcP-LPS, AuNP-OmpW-LPS, or AuNP-hemagglutinin-LPS resulted in 50-80% survival (**Table 1**) (101). Analysis of humoral immune responses showed that serum from mice immunized with OpcP- and OmpW-formulations exhibited high LPS- and protein-specific IgG2c levels, indicating a Th1-biased (IgG2c > IgG1) immune response. The immune serum was associated with enhanced macrophage-mediated phagocytosis of *B. mallei* ATCC 23344 and reduced bacterial adherence to murine lung epithelial cells (101).

The effectiveness of different AuNP-protein-LPS candidates against *B. pseudomallei* has also been evaluated in C57BL/6 mice (102). Specifically, mice immunized with AuNP-OpcP-LPS or AuNP-OpcP1-LPS demonstrated 90% and 30% protection at day 35, respectively, against a 6 LD_50_ intranasal challenge of *B. pseudomallei* K96243 (**Table 1**). Upon initial experimentation, the combination of AuNP-OpcP-LPS and AuNP-OpcP1-LPS were further evaluated and deemed to be the most effective, with 100% survival at day 35 post-challenge with 5 LD_50_ of *B. pseudomallei* K96243 (**Table 1**). Most of the surviving mice had low bacterial loads in lungs, livers, and spleens with only a few pathological lesions. AuNP-OpcP-LPS, AuNP-OpcP1-LPS, and AuNP-OpcP-LPS/AuNP-OpcP1-LPS elicited robust LPS- and protein-specific IgG responses which promoted macrophage uptake of *B. pseudomallei* K96243. Additionally, immunized mice demonstrated high levels of LPS- and protein-specific IgG and IgA in their lungs as well as mixed Th1 and Th17 biased protein-specific cytokine responses upon splenocyte re-stimulation (102).

### 2.5 Virus-like particle vaccines

Virus-like particles (VLPs) are protein-based nanoparticles that are frequently used as carriers in conjugate vaccine platforms and for delivering immunotherapies (103). These particles are non-infectious due to lack of genetic material necessary to replicate but may be engineered to express immunogenic antigens that elicit robust B cell responses (104). A recent study employed the external decoration approach by displaying Hcp1 protein on the surface of P22 VLPs (105). Mice immunized with conjugated Hcp1-VLPs demonstrated robust Hcp1-specific IgG, IgG1, IgG2c, and IgA titers, irrespective of low (5 μg) or high (10 μg) doses of Hcp1-VLPs, compared to mice that received PBS or unconjugated VLPs as controls. The serum obtained from Hcp1-VLPs immunized mice enhanced antibody responses and promoted phagocytosis of opsonized bacteria by macrophages (105). Future animal challenge studies are needed to evaluate the protective capacity of Hcp1-VLPs against *B. pseudomallei*.

### 2.6 DNA vaccines

Plasmid-based DNA vaccines are designed to deliver genes encoding specific antigens that can induce humoral and cellular immune responses against pathogens and are considered cost-effective and amenable to large-scale manufacture (106–109). Recently, a *B. pseudomallei* flagellin (FliC) plasmid DNA vaccine, pVAX-hTPA-FliC, was evaluated in C57BL/6 mice using either a rapid dermal tattoo or an intranasal delivery system. Following an intranasal challenge with *B. pseudomallei* 1026b, a single intranasal immunization with pVAX-hTPA-FliC was more successful than dermal tattoo delivery in reducing bacterial loads, pulmonary cytokine levels (TNF-α, IL-6, CXCL1), plasma cytokine levels (TNF-α, IL-6, IFN-γ), lung pathology scores, systemic inflammation, and organ damage. However, a single intranasal immunization failed to elicit detectable anti-FliC IgG responses. Results demonstrated that only 53% survival was observed in mice receiving intranasal immunization with pVAX-hTPA-FliC at 14 days post-challenge with *B. pseudomallei* 1026b (**Table 1**) (110). As DNA vaccines against *B. pseudomallei* have not been previously explored, future studies are needed to focus on the optimization of vaccine formulations and routes of administration to provide robust humoral and cellular immune responses needed for protection against melioidosis.

### 2.7 Viral vector-based vaccines

Viral vector-based vaccines are designed to deliver genes encoding specific antigens into host cells (111). These types of vaccines can elicit immune responses without the need for an adjuvant and are amenable to large-scale and cost-effective production (112, 113). Viral vector-based vaccine platforms have been developed for immunization against melioidosis and glanders using Parainfluenza virus 5 (PIV5) as the vector to deliver the conserved *B. mallei* autotransporter protein BatA (PIV5-BatA) to target cells. Following an inhalational challenge with 5 LD_50_ *B. pseudomallei* K96243, BALB/c mice immunized with a single intranasal dose of PIV5-BatA displayed survival rates of 80% on day 10 and 60% day 35 (**Table 1**) (114). Of the surviving mice, 78% and 44% had no culturable bacteria in their lungs and spleen, respectively. Additionally, PIV5-BatA provided 84% survival at day 10 and 74% survival at day 40 against an inhalational challenge with *B. mallei* ATCC 23344 (**Table 1**) (114). Analysis of immune responses revealed that BatA-specific IgG and IFN-γ-secreting T cell responses were critical for providing protection (114). While the PIV5-BatA vaccine candidate showed significant promise for providing immunity against *B. pseudomallei* and *B. mallei*, further studies will be needed to evaluate the longevity of protection and the need for booster doses, which could influence the overall efficacy of this platform in preventing melioidosis and glanders.

## 3 Conclusions and future directions

Since *B. pseudomallei* is a facultative-intracellular bacterium, it is anticipated that protective immunity against this pathogen will be complex. Several studies support that humoral immune responses are important for controlling early stages of an infection (extracellular phase) whereas cellular immune responses are important for controlling later stages of an infection (intracellular phase). It is expected, therefore, that a vaccine that elicits both types of responses will be required to provide full protection against disease. This review summarizes the various approaches that have been used to develop melioidosis vaccine candidates over the past seven years. While significant progress has been made in this area, the development of a broadly effective vaccine continues to be challenging (60, 115–118). Several factors are likely responsible for this including that *B. pseudomallei* 1) is a highly virulent pathogen that requires specialized permissions, facilities and containment practices to be studied, 2) expresses an impressive array of virulence factors that enables it to survive and replicate in a variety of different cell types and tissues and 3) exhibits a multifaceted lifestyle that enables it to avoid clearance by host immune defenses.

At present, mouse models remain the primary means for evaluating melioidosis vaccine efficacy, with BALB/c and C57BL/6 mice commonly being used for acute and chronic infection studies, respectively. Using these models, several live-attenuated, glycoconjugate-based and/or protein-based subunit, OMV, nanoparticle-based, and viral vector-based vaccine candidates have yielded promising results. Robust protection against lethal doses of *B. pseudomallei* have been observed with some of these vaccines, however, sterilizing immunity has proven difficult to achieve especially during protracted challenge studies. There is strong evidence to support that high titer, opsonizing IgG responses specific for *B. pseudomallei* CPS are critical for controlling early stages of infection (48). Furthermore, there appears to be a correlation with the most promising melioidosis vaccine candidates and their ability to stimulate robust Th1- and Th17-like humoral and cellular immune responses. Such observations are consistent with studies demonstrating that melioidosis patient survival correlates with strong IFN-γ secreting T cell responses against *B. pseudomallei* protein antigens (68). Further studies are required, however, to better establish correlates of antigen-induced immunity to guide the rational design of future melioidosis vaccines.

Newer technologies, including mRNA vaccines and the use of in silico methodologies to guide the design of multi-epitope-based peptide vaccines, may also represent novel approaches for immunization against melioidosis (119–121). The benefits of these platforms include low production costs, scalability, and the ability to induce robust humoral and cellular immune responses. A limitation of both approaches, however, is their inability to express non-protein antigens, specifically polysaccharides, which have been proven to be important components of several vaccine platforms described in this review. To address this issue, it will be important to identify and use proteins or B cell epitopes that can stimulate protective opsonizing and complement-activating immune responses, similar to those elicited by OPS and CPS antigens.

Moving forward, efforts should be placed on 1) defining specific correlates of immunity associated with efficacious vaccines, 2) investigating how different adjuvants and immune-modulators can be used to potentiate protective immune responses, 3) optimizing dosing and routes of immunization and 4) the use of immunocompromised mouse models (e.g. diabetic mice). Although mice have been invaluable for pre-clinical evaluation of melioidosis vaccines, studies using higher-order animal species (e.g. NHPs) will likely also be necessary to assess the safety and immunogenicity of lead vaccine candidates prior to their advancement into human clinical trials.

An effective vaccine aimed at reducing the incidence and severity of melioidosis in endemic regions would be predicted to improve morbidity and mortality rates as well as decrease healthcare costs. Individuals who are vaccinated may have a lower risk of developing severe symptoms or complications and have a reduced need for prolonged antibiotic therapy (122). Effective vaccine candidates should be considered for their ability to generate protective immunity in high risk populations such as individuals with diabetes, chronic lung or kidney disease, thalassemia, other immunocompromising conditions and the elderly (123). Furthermore, future studies should not only focus on safety, immunogenicity, durability and efficacy but also consider stability, cost-effectiveness and accessibility for use in endemic regions worldwide (60, 115, 116).

To date, good progress has been made by the few research groups that have taken on the challenge of developing a safe, affordable, and efficacious melioidosis vaccine. Based on recent successes, the melioidosis research community is optimistic that this can be achieved but also acknowledges that significant obstacles must be overcome for this to happen. Unless funding agencies, public health officials, and government policymakers recognize the true burden of melioidosis in countries where it is endemic, and implement strategies to combat this disease, a licensed vaccine will remain elusive.
